# Congenital Anomalies and Associated Factors among Newborns in Bishoftu General Hospital, Oromia, Ethiopia: A Retrospective Study

**DOI:** 10.1155/2021/2426891

**Published:** 2021-03-31

**Authors:** Samuel Gedamu, Endalew Gemechu Sendo, Workinesh Daba

**Affiliations:** ^1^Department of Midwifery, College of Health Sciences, Assosa University, Assosa, Ethiopia; ^2^School of Nursing and Midwifery, College of Health Sciences, Addis Ababa University, Addis Ababa, Ethiopia

## Abstract

**Background:**

Congenital anomalies affect millions of babies worldwide with prevalence of 3%, and it is estimated that, globally, 303,000 newborns die within the first 4 weeks of life due to this problem.

**Objective:**

This study aimed to assess congenital anomalies and their associated factors among newborns in Bishoftu General Hospital, Oromia Regional State, Ethiopia. *Setting*. Bishoftu General Hospital, Oromia, Ethiopia. *Study Design *. A retrospective cross-sectional study was employed. *Participants*. All birth records from September 14, 2018, to March 14, 2019, were reviewed. A census method was applied for this study. The data were collected from birth registration books through structured checklist. We used Statistical Package for the Social Sciences (SPSS) version 24.0 for data analysis. Crude and adjusted odds ratio with 95% confidence interval was computed. Statistical significance was set at *P* < 0.05.

**Result:**

Out of 2,218 live births, 23 newborns were diagnosed with congenital malformations, making the prevalence rate of 1% (i.e., 10/1000 live births in the specified time period). Maternal age above 35 years (AOR = 6.5; 95% CI = 2.4–18), birth order above 3 (AOR = 8.4; 95% CI = 3.4–20.7), birth weight less than 2.5 kg (AOR = 0.3; 95% CI = 0.1–0.9), and singleton pregnancy (AOR = 6.4; 95% CI = 2–18.9) had a significant association with the incident of congenital anomalies, while iron folate use before and/or during early pregnancy and urban residence (AOR = 0.3; 95% CI = 0.1–1) had a protective effect against congenital anomalies (AOR = 0.036; 95% CI = 0.008–0.15).

**Conclusion:**

The findings of this study showed that there is a burden of congenital anomalies in the study area. Sustainable surveillance and registry systems are thus required for intervention programs and it is crucial to include them under Ethiopian demographic health survey (EDHS) report.

## 1. Introduction

Birth defects or congenital anomaly can be defined as structural or functional abnormalities, comprising metabolic disorders, which are usually presenting from childbirth [1]. Birth defects can cause spontaneous abortions and stillbirths; nonetheless, these are not recognized as causes of mortality and disability among infants and children below five years of age. They can, however, be life-threatening, result in long-term disability, and negatively affect individuals, families, healthcare systems, and societies [1, 2]. Congenital anomalies affect millions of babies worldwide, affecting 3% of babies born. Some birth defects are more severe than others, and it is estimated that globally, 303,000 newborns die within the first 28 days of life due to congenital anomalies [3]. According to World Health Organization (WHO), 17%–42% of infant mortality was attributed to congenital anomalies [4]. For instance, in 11 European Registration of Congenital Anomalies and Twins (EUROCAT) countries, average infant mortality with congenital anomaly was reportedly 1.1 per 1000 births, with higher rates where termination of pregnancy for fetal anomaly (TOPFA) is illegal. The rate of stillbirths with congenital anomaly was 0.6 per 1000 and the average TOPFA prevalence was 4.6 per 1000, nearly three times more prevalent than stillbirths and infant deaths combined [5].

In Spain, there were 13,660 deaths (53.4% males and 46.6% females) due to rare CAs during the period of 1999–2013. In terms of type of CA, the highest percentage (40.3%) of deaths corresponded to rare CAs of the circulatory system, followed by 16.9% due to chromosomal abnormalities, 14.5% due to other congenital malformations, and 9.2% due to rare CAs of the nervous system [6].

According to the current Ethiopian Demographic Health Survey 2016 [7], the infant mortality rate was reported to be 48 deaths per 1,000 live births. The same survey reported that the child mortality rate was 20 deaths per 1,000 children surviving to the age of 12 months, while the overall under-5 mortality rate was 67 deaths per 1,000 live births. The neonatal mortality rate was 29 deaths per 1,000 live births, and the postneonatal mortality rate was 19 deaths per 1,000 live births; however, the potential causes for these deaths were not described in this survey, which might be congenital anomaly among others.

According to the study conducted in the city of Sao Paolo, maternal age, multiple pregnancies, and low birth weight are associated with the presence of congenital anomalies, and female gender was considered as protective for the occurrence of CAs [8]. Another study that was conducted in Pakistan's Mardan Medical Complex showed that consanguinity and lack of folic acid intake are the most common risk factors for the presence of CAs [9].

The study conducted in Latvia on epidemiological aspects of congenital anomalies and associated risk factors indicates that newborns born from multiple pregnancies, male neonate, preterm birth, maternal alcohol consumption and smoking, not having antenatal care, and mothers' age above 35 are highly associated with the presence of CAs [10].

According to a study in Tanzania, maternal factors such as lack of periconceptional use of folic acid and an inadequate attendance to antenatal clinic and infant factors such as female sex, a birth weight of 2.5 kg or more, singleton pregnancy, and a birth order above 4 were significantly associated with CAs [2].

In Ethiopia, limited studies have been conducted as regards risk factors associated with congenital anomalies. Hence, this study aimed to assess congenital anomalies and their associated factors among newborns in Bishoftu General Hospital, Oromia Regional State, Ethiopia.

## 2. Methods

### 2.1. Study Area and Period

Bishoftu General Hospital was purposively selected based on its case load for this study. It is found in Bishoftu town, which is located in Oromia Regional State, East Shewa Zone, at a distance of 47 km from Addis Ababa. According to 2015 Central Statistics Agency, it is estimated that the total population of Bishoftu is 147,100 and the town has one general hospital and three health centers. The hospital was established in 1948 to offer services for 1.2 million people. Monthly report of delivery in Bishoftu General Hospital ranges from 350 to 400. The study was conducted from March 15 to April 30, 2019.

*Study design*. A retrospective cross-sectional study was employed.

*Source population*. It included all birth records of newborns who were delivered in Bishoftu General Hospital.

*Study population*. The study's population was all neonates born and registered in Bishoftu General Hospital from September 14, 2018, to March 14, 2019.

### 2.2. Sampling Methods

A census method was applied for this study. All birth records from September 14, 2018, to March 14, 2019, were reviewed. Newborns diagnosed with at least one birth defect were included in the study, while records with incomplete information (those records that miss more than two variables) and lost cards were excluded from the study.

### 2.3. Instrument

The data were collected from birth registration books through structured checklist adapted from previous literature [3, 11]. The checklist contains sociodemographic characteristics, types of congenital anomaly, and factors associated with congenital anomaly (i.e., maternal factors and infant factors). In this study, the primary outcome variable was congenital anomaly. It refers to any observable abnormality of structure or body part presenting at birth or any birth defect recorded on the chart. The covariates included in the study were sociodemographic characteristics (age, residence, marital status, and ethnicity) and presence of maternal factors (ANC visit, folic acid use, smoking, alcohol use, chronic illness, and infectious disease) and fetal factors (sex, birth order, gestational age, pregnancy type, and birth weight). Two B.S. midwives were assigned to extract the data, and a Principal Investigator and one Senior B.S. midwife supervised the process of data collection.

### 2.4. Data Quality Control

Measures were taken to ensure the quality of collected data. The checklist was pretested before actual study and necessary changes were then made to maximize the reliability of the data. Likewise, we maintained confidentiality and anonymity of the study. Lastly, the data were checked for completeness by the principal investigator.

### 2.5. Data Analysis

We used Statistical Package for the Social Sciences (SPSS) version 24.0 for data analysis. Logistic regression model was used to examine the association between factors and congenital anomalies. Categorical variables were summarized as proportions and were compared using Pearson's Chi square test. Crude and adjusted odds ratio with 95% confidence interval was computed. Variables with *P* < 0.02 on binary logistic regression were included in multiple logistic regressions. Statistical significance was set at *P* < 0.05.

### 2.6. Patient and Public Involvement

No patients were involved.

## 3. Results

### 3.1. Sociodemographic Characteristics

A total of 2,218 delivery charts were reviewed. The majority (93.8%) of mothers were married and their mean age was 25.7 (ranges from 16 to 45 years). About 2/3 of the infants were females and, regarding gestational age at the time of delivery, more than 3/4 of infants were born at term. The majority (87.8%) of births were singleton (Table 1).

### 3.2. Prevalence of Common Congenital Anomalies

**Figure 1:**
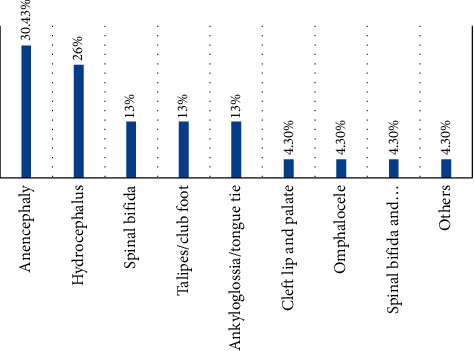
Frequency distribution of the most prevalent types of congenital anomalies in Bishoftu General Hospital, Oromia, Ethiopia, 2019.

Out of 2,218 live births, 23 newborns were diagnosed with congenital malformations, making the prevalence rate of 1% (i.e., 10/1000 live births in the specified time period). From a total of birth defects, the two most common congenital anomalies found in this study were anencephaly, 7 (30.4%), and hydrocephalus, 6 (26.1%), respectively (Figure 1).

### 3.3. Factors Associated with Congenital Anomalies

Tables 2 and 3 depict multivariate analysis of maternal and infant factors associated with congenital anomalies in this study. Maternal age above 35 years (AOR = 6.5; 95% CI = 2.4–18), birth order above 3 (AOR = 8.4; 95% CI = 3.4–20.7), birth weight less than 2.5 kg (AOR = 0.3; 95% CI = 0.1–0.9), and singleton pregnancy (AOR = 6.4; 95% CI = 2–18.9) had a significant association with the incident of congenital anomalies, while iron folate use before and/or during early pregnancy (AOR = 0.036; 95% CI = 0.008–0.15) and being from urban area (AOR = 0.3; 95% CI = 0.1–1) had a protective effect against congenital anomalies.

## 4. Discussion

The objective of this retrospective cross-sectional study was to report on the prevalence and factors associated with congenital anomalies among newborns in Bishoftu General Hospital, Oromia, Ethiopia, 2019. The prevalence of congenital anomalies in this study was 1%, which means 10/1000 live births, which is near to the finding (19 per 1000) of a study conducted in central and southwest Ethiopia by Taye et al. [12]. The observed similarity in prevalence with long-range retrospective studies, which are not similar to this study, is difficult to explain. However, the fact that both studies were done in hospitals with reviewing medical records may offer some explanation for the observed similarities.

This study is also comparable with a study conducted in Nigeria, which had prevalence of 2.8%, even though the study was conducted on the newborns who were admitted in neonatal intensive care unit (NICU) [13]. In addition to this study, there are various studies showing that the prevalence rate of congenital anomalies ranges from 1.23 to 3%, which is almost similar to the finding of this study [8, 9, 13]. But the finding of this study is lower than that of a study conducted in Mwanza, Tanzania, by Florentine Mashuda et al., which had a prevalence rate of 29% [2]; the observed difference is maybe due to the study population, as they used neonates admitted in hospitals, while we used all neonates who were born in the stated hospital.

In this study, the highest proportion of congenital anomalies was anencephaly (30.4%), followed by hydrocephalus (26.1%) and spinal bifida (8.7), and omphalocele (4.3%) was the least prevalent type of CAs. So this study is almost similar to a study conducted in Mardan Medical Complex, Pakistan, which reported hydrocephalus (27.3%) and anencephaly (18%) as the most prevalent types and omphalocele (1.7%) as the least prevalent type [9]. It is also near to a study conducted in Ethiopia, which ranks anencephaly at the third place and spinal bifida at first place [12]. But the finding of this study is in contrast with a study conducted in Southeast Nigeria, which reported cleft lip/palate as the most prevalent type of congenital anomalies [13]. It is also in contrast with a study conducted in Brindisi, Italy, and Mount-Lebanon, Lebanese, Latvia, which reported congenital heart defects as the most prevalent type of congenital anomalies [10, 14, 15]. The observed difference is maybe due to the difference in geographical area.

The finding of this study shows that maternal age above 35 years (AOR = 6.5; 95% CI = (2.4–18); *P* value = 0.001), which means mothers with age above 35 years, had 6.5 times greater chance of having congenitally deformed babies than the mothers with age below 35 years and urban residence (AOR = 0.3; 95% CI = 0.1–1; *P* value = 0.043), and maternal use of folic acid (AOR = 0.036; 95% CI = .0.008–0.15; *P* value =< 0.001) is protective against CAs. This study is similar to a study conducted in Tanzania, which showed that maternal age above 35 years and no use of folic acid during pregnancy are associated with the occurrence of CAs [2]. This finding is also similar to that of a study conducted in central and southern parts of Ethiopia, which showed that folic acid intake during pregnancy is protective against the occurrence of congenital anomalies [3].

There are various studies showing that maternal alcohol intake, smoking history, chronic illness, and history of infectious disease had significant association with the occurrence of congenital anomalies [3, 8, 15], but the finding of this study showed that there was no association between these variables and occurrence of congenital anomalies. The difference may be due to the methodology they employed and, hence, this study is a retrospective study that uses secondary data, and this may result in a difference.

On the side of infant factors associated with congenital anomalies, according to the finding of this study, birth order above 3 (AOR = 8.4; 95% CI = 3.4–20.7; *P* value = 0.001), which indicates that infant who was delivered third or above, had 8.4 times chance of being a congenitally anomalous baby than those who were delivered second or below, and birth weight less than 2.5 kg (AOR = 0.3; 95% CI = 0.1–0.9; *P* value = 0.037) and singleton pregnancy (AOR = 6.4; 95% CI = 2–18.9; *P* value = 0.001) had significant association with the presence of congenital anomalies. The finding of this study is similar to that of a study conducted in Mwanza, Tanzania, which showed that singleton pregnancy and birth order of 3 and above had significant association with presence of congenital anomalies [2]. But the finding of this study is in contrast with that of a study conducted in the city of Sao Paolo by Cosme et al., which reported that premature babies and twins had significant association with occurrence of CAs [8]. The observed difference is maybe due to the time period, as the study conducted in the city of Sao Paolo was a four-year retrospective study.

### 4.1. Strengths and Limitations of This Study

This study is the first aiming to assess congenital anomalies and their associated factors among newborns in Oromia Regional State, Ethiopia, which adds new evidence about positive and negative predictors for congenital anomalies from this region, where scientific evidence is limited. This would be a valid reason to publish this study. However, this cross-sectional study can only be generalized to this cohort of women/neonates in the study setting. This cross-sectional study also cannot determine cause-and-effect relationship. Another limitation is that this study is a retrospective study, where hospital-based birth records of cases were used.

## 5. Conclusion

Maternal age above 35 years, birth order above 3, birth weight less than 2.5 kg, and singleton pregnancy had a significant association with the occurrence of congenital anomalies, while iron folate use before and/or during early pregnancy and urban residence had a protective effect against congenital anomalies. The findings of this study showed that there is an increasing burden of congenital anomalies in the study area. Sustainable surveillance and registry systems are thus required for intervention programs. In this study, the percentage of women not taking folic acid supplements in early pregnancy was substantial. Hence, efforts should be made to guarantee that more women utilize folic acid during pregnancy and/or before conception for it has been proven by a number of authors to reduce incidence of some congenital anomalies.

## Figures and Tables

**Table 1:** Frequency distribution of sociodemographic characteristics of the mother and infant factors in Bishoftu General Hospital, Oromia, Ethiopia, 2019.

	Frequency (*n* = 2,218)	Percent
*Marital status*		
Married	2081	93.8
Living together	26	1.2
Never married	1	.0
Divorced/separated	110	5

*Residence of the mother*		
Urban	1530	69.0
Rural	688	31.0

*Sex of the infant*		
Male	820	37.0
Female	1398	63.0

*G/A at the time of delivery*		
Preterm	268	12.1
Term	1816	81.9
Postterm	134	6.0

*Pregnancy type*		
Twins	270	12.2
Singleton	1948	87.8

**Table 2:** Multivariate analysis of maternal factors associated with congenital anomalies in Bishoftu General Hospital, Oromia, Ethiopia, 2019.

Variable	Category	Presence of CAs		COR (95% CI)	*P* value	AOR (95% CI)	*P* value
		Yes	No				
Age of the mother	1 ≥ 35	11 (8.6%)	117 (91.4%)	0.06 (0.03–0.14)	0.001	6.5 (2.4–18)	<0.001
	2 < 35	12 (0.6%)	2078 (99.4%)				
Residence	Urban	8 (0.5%)	1522 (99.5%)	4.2 (1.8–10)	0.001	**0.3** (**0.1–1)**	**0.043**
	Rural	15 (2.1%)	673 (97.8%)				
ANC visit	Yes	10 (0.5%)	1885 (99.5%)	7.9 (3.4–18.2)	0.001	3.2 (0.8–12.8)	0.1
	No	13 (4%)	310 (96%)				
Maternal use of folic acid	Yes	5 (0.27%)	1823 (99.7%)	17.6 (6.5–47.8)	0.001	**0.036** (**0.008–0.15)**	**0.001**
	No	18 (4.6%)	372 (95.4%)				
Maternal alcohol intake	Yes	1 (1.5%)	64 (98.5%)	0.66 (0.08–4.97)	0.688	—	—
	No	22 (1%)	2131 (99%)				
Smoking history	Yes	2 (2.1%)	92 (97.9%)	0.45 (0.1–2)	0.298	—	—
	No	21 (1%)	2102 (99%)				
History of chronic illness	Yes	4 (6.8%)	55 (93.2%)	0.12 (0.04–0.37)	0.001	2.2 (0.54–8.8)	0.26
	No	19 (0.9%)	2140 (99.1%)				
History of infectious disease	Yes	4 (2%)	197 (98%)	0.468 (0.158–1.4)	0.2	—	—
	No	19 (0.9%)	1998 (99.1%)				

**Table 3:** Multivariate analysis of infant factors associated with congenital anomalies in Bishoftu General Hospital, Oromia, Ethiopia, 2019.

Variable	Category	Presence of CAs		COR (95% CI)	*P* value	AOR (95% CI)	*P* value
		Yes	No				
Sex of the infant	Male	6 (0.73%)	814 (99.26%)	1.67 (0.65–4.2)	0.28	—	—
	Female	17 (1.2%)	1381 (98.8%)				
Birth order	≥3	12 (2.8%)	416 (97.2%)	0.2 (0.09–0.5)	0.001	**8.4** (**3.4-20.7%)**	**0.001**
	<3	11 (0.6%)	1779 (99.4%)				
Birth weight	≥2.5	14 (0.7%)	1927 (99.3%)	4.6 (1.98–10.78)	0.001	**0.3** (**0.1–0.9)**	**0.037**
	<2.5	9 (3.2%)	268 (96.8%)				
Gestational age at the time of delivery	Preterm	3 (1.1%)	265 (98.9%)	0.66 (0.68–6.4) 0.7 (0.7–5.3)	0.9	—	—
	Term	19 (1%)	1797 (99%)				
	Postterm	1 (0.75%)	133 (99.25%)				
Pregnancy type	Twin	11 (4%)	260 (96%)	0.14 (0.06–0.35)	0.001	**6.4** (**2–18.9)**	**0.001**
	Singleton	12(0.6%)	1935(99.4%)				

## Data Availability

All data generated or analyzed during this study are included in the manuscript.

## Abbreviations

AOR: Adjusted odds ratio
CI: Confidence interval
CAs: Congenital anomalies
TOPFA: Termination of pregnancy for fetal anomaly
EUROCAT: European Registration of Congenital Anomalies and Twins.
